# Emergence of Inequality in Income and Wealth Dynamics

**DOI:** 10.3390/e25081129

**Published:** 2023-07-27

**Authors:** Changhee Cho, Jihun Park, Biseko Juma Mafwele, Quang Anh Le, Hye Jin Park, Jae Woo Lee

**Affiliations:** 1 Department of Physics, Inha University, Incheon 22212, Republic of Korea; 2 Institute of Advanced Computational Sciences, Inha University, Incheon 22212, Republic of Korea

**Keywords:** wealth inequality, poverty, income distribution, power-law, scaling, saving, debt, tax

## Abstract

Increasing wealth inequality is a significant global issue that demands attention. While the distribution of wealth varies across countries based on their economic stages, there is a universal trend observed in the distribution function. Typically, regions with lower wealth values exhibit an exponential distribution, while regions with higher wealth values demonstrate a power-law distribution. In this review, we introduce measures that effectively capture wealth inequality and examine wealth distribution functions within the wealth exchange model. Drawing inspiration from the field of econophysics, wealth exchange resulting from economic activities is likened to a kinetic model, where molecules collide and exchange energy. Within this framework, two agents exchange a specific amount of wealth. As we delve into the analysis, we investigate the impact of various factors such as tax collection, debt allowance, and savings on the wealth distribution function when wealth is exchanged. These factors play a crucial role in shaping the dynamics of wealth distribution.

## 1. Introduction

The distribution of wealth or income has become a prominent subject in economics and econophysics, drawing increased attention to the global income inequality issue and potential policy solutions. According to Pareto’s law, approximately 20% of the population possesses about 80% of the total societal wealth. Pareto also observed that the distribution function of individual income in Italy conforms well to the power law (Pareto, 1897) [1]. Gibrat, on the other hand, proposed that income follows a lognormal distribution, assuming multiplicative stochastic processes for income changes (Gibrat, 1931) [2]. Mandelbrot introduced the Pareto–Levy law of income distribution, noting that the Pareto–Levy Markovian process can be approximated by a random walk of the logarithm of income for higher income ranges [3]. Recent research by Piketty and Saez reveals that wealth inequality has been progressively widening in the United States and Europe since 1950 [4].

Angle (1986) investigated the distribution function of individual wealth [5]. It was found that size distributions of wealth across societies with varying levels of technology can be effectively represented by a family of gamma distributions [6]. Lux introduced Angle’s pioneering work in the field of econophysics [7]. However, the analysis of wealth distribution poses challenges due to the scarcity of personal wealth data. Although the available wealth data exhibit qualitative similarities to income data, they demonstrate a lower exponent in the tail distribution, indicating higher levels of inequality [8]. In a study by Souma, the Japanese personal income distribution was analyzed for 112 years in the high-income range and 44 years in the middle-income range [9]. The findings revealed that the income distribution followed a log-normal distribution with a power-law tail, but it exhibited variations from year to year. Aoyama et al. examined income and income tax distribution data for individuals in Japan during the fiscal year 1998, as well as the distribution of debts owed by bankrupt companies from 1997 to March 2000. Their analysis indicated that both datasets follow a power law, with a Pareto exponent close to −2 based on rank-size plots [10].

Dragulescu and Yakovenko examined wealth and income distribution data obtained from the Bureau of Census in the United Kingdom and the International Revenue Service in the United States. Their findings revealed that most of the population could be characterized by an exponential distribution, while the upper tail of the distribution followed a power law pattern [11,12]. Similarly, Banerjee et al. (2006) conducted an analysis of personal income distribution data obtained from the Australian Bureau of Statistics. They concluded that an exponential function provides a suitable description for approximately 98% of the population in the lower segment of the distribution [13].

The analogy between the exchange of money in economic activity and the exchange of energy in the physical impact of gas molecules has been explored by some statistical physicists. The field of econophysics suggests that the distribution of wealth is influenced by the exchange of wealth or income between economic agents, much like the exchange of energy between gas molecules during collisions in the kinetic theory of gases. Just as the collision of gas molecules leads to an equilibrium state, the exchange of money in an economic system contributes to the distribution of income.

Recently, several models have been proposed that focus on pairwise transactions resulting in money transfers between individuals. These models operate under the assumption that the total amount of money in the economy remains constant, similar to the role of energy conservation in statistical mechanics. However, these kinetic exchange models have faced criticism from economists. They argue that the models oversimplify the real-world scenario, as money is not conserved and the dynamics of economic systems are more complex than what these models account for [14,15,16,17,18].

In a study by Ispolatov et al., the asset exchange model was introduced, resulting in a power-law distribution of wealth in the greedy multiplicative exchange model [19]. Bouchaud and Mezard introduced a model that encompasses both the exchange of wealth between individuals and random speculative trading [20]. Their observations indicated that the distribution of wealth followed Pareto behavior. Dragulescu and Yakovenko introduced a straightforward model for money transfers between two agents [21]. In their study, they observed that the distribution of money in the stationary state followed the Boltzmann–Gibbs distribution. Additionally, they investigated a kinetic money exchange model that incorporates debt, and they found that the distribution of money still adhered to the Boltzmann–Gibbs distribution. Chakraborti and Chakrabarti presented a simple kinetic model for money exchange that includes a saving mechanism [22]. In their model, the total amount of money is conserved, and the number of agents remains constant. When the agents do not have any savings, the stationary distribution of money aligns with Gibbs’ distribution.

In the simple kinetic money exchange model, the distribution function of money can be described by an exponential function. When incorporating debt into the model, the distribution function remains largely unchanged. However, in the model where agents save a portion of their money and exchange the rest, the tail of the money distribution function follows a power-law distribution. The introduction of wealth distribution and the kinetic exchange model of money has sparked significant attention in the field of econophysics [12,13,14,15,16,17,18,19,20,21,22,23,24,25,26,27,28,29,30,31,32,33,34,35,36,37,38,39,40,41,42,43]. In this review, we aim to provide an overview of the current developments in kinetic exchange models and the distribution of income and wealth.

## 2. Wealth and Income Distribution

With the accumulation of data on income and national wealth, there has been an increase in research and interest in wealth distribution. Alfred Pareto published a study revealing that 20% of the population owned 80% of the land in Italy, now referred to as the Pareto Principle or 80–20 Rule [44]. The Pareto law is observed when the wealth distribution function follows the power law with respect to the size of wealth, determining the distribution of people who own wealth [1,45],

(1) $$p\left(w\right)\sim w^{-\left(1+\alpha \right)}.$$

where the Pareto index *α*, which characterizes the wealth distribution, is not universal and exhibits different values across countries. According to Newman, when *α* is equal to 2.1, the top 20% of the population possesses 86% of the total wealth [45]. This phenomenon is often referred to as the Matthew effect, as described by Gladwell [46]. The distribution of wealth, following a power law similar to the Pareto distribution, leads to a slow decrease in the distribution function, determined by the exponent α. Consequently, there is a substantial accumulation of wealth at the tail end of the distribution function, known as the long-tail or heavy-tail distribution.

Gibrat’s research revealed that the Pareto law is applicable primarily in the high-income range, while the distribution of income in the middle-income range follows a log-normal distribution function [2]. Furthermore, Dragulescu and Yakovenko found that the distribution function of individual income in the United States adheres to an exponential distribution [11,12]. Depending on the income range, a wide range of income data can exhibit either an exponential or power law distribution [11,12,47,48,49,50,51,52,53].

Income and wealth are distinct concepts in economics. Income refers to the amount of money a person earns during a specific period, such as their early income. On the other hand, wealth represents the total value of assets a person owns, including money, assets, and property. When examining the dynamics of money distribution, we involve the distribution function related to income amounts. However, when analyzing wealth distribution, the distribution function pertains to the total wealth of an individual.

The study of wealth distribution in the field of econophysics originated in the late 1990s with the analysis conducted by Levy and Solomon on the income distribution of the top 400 richest Americans, which was published in Forbes in 1996 [49]. Their report revealed that the distribution of wealth among the wealthy follows a power law with an exponent of α = 1.36. This distribution with a fat tail was remarkably similar to the power law distribution observed in stock price fluctuations in the stock market, as observed by Mantegna and Stanley in 1995 [50]. Theoretical derivation of the wealth distribution function in the asset exchange model was performed by Ispolatov et al., demonstrating that the distribution of wealth in the greedy multiplicative exchange follows a power function distribution [19].

The cumulative distribution function of wealth, denoted as $P\left(w\right)$, can be defined as the integral from w to infinity of the probability density function as $P\left(w\right)=\int_{w}^{\infty }p\left(w\right)dw$. The complementary cumulative wealth distribution follows a power-law behavior, $P\left(w\right)\sim w^{-\alpha }$ where α is the exponent of power law. When examining income distribution of a country, different functional structures are observed. For instance, in Japan, the income distribution function for individuals exhibits a power-law tail [9,10]. This power-law distribution has also been observed in various countries, including the United States, the United Kingdom, Italy, Austria, and Brazil [11,13,14]. However, the middle-income class does not follow a power-law distribution. Gibrat proposed that the income distribution function for the middle class follows a lognormal distribution, described by a specific formula [2].

(2) $$p\left(w\right)=\frac{1}{w\sqrt{2\pi \sigma^{2}}}exp\left[-\frac{ln^{2}\left(w/\bar{w}\right)}{2\sigma^{2}}\right],$$

where $\bar{w}$ is the mean and $\sigma^{2}$ is the variance. The Gibrat index is $\beta =1/\sqrt{2\sigma^{2}}$, and the smaller this value, the greater the inequality in the income distribution.

Montroll and Shlesinger conducted an analysis of the income distribution in the United States from 1935 to 1936, revealing that the majority of income ranges followed a lognormal distribution, while the top 1% of the highest income bracket exhibited a Pareto tail distribution [51,52]. In a study examining individual incomes in both the United States and the United Kingdom, Dragulescu and Yakovenko discovered that the income distribution function within the lower-income range followed an exponential function instead of a lognormal distribution. However, the high-income range demonstrated a power-law distribution [11,12,21,53,54,55,56,57]. More specifically, if an individual’s income falls below $w<w_{c}$, the income distribution function can be described using the following formula:

(3) $$p\left(w\right)\sim e^{-\frac{w}{T_{w}}},$$

where $T_{w}$ is the effective wealth temperature.

In general, the income distribution function is expressed by the double distribution function of the exponential distribution and the power law as

(4) $$p\left(w\right)\sim w^{-\left(1+\alpha \right)}e^{-\frac{w}{T_{w}}}.$$

**Figure 1:**
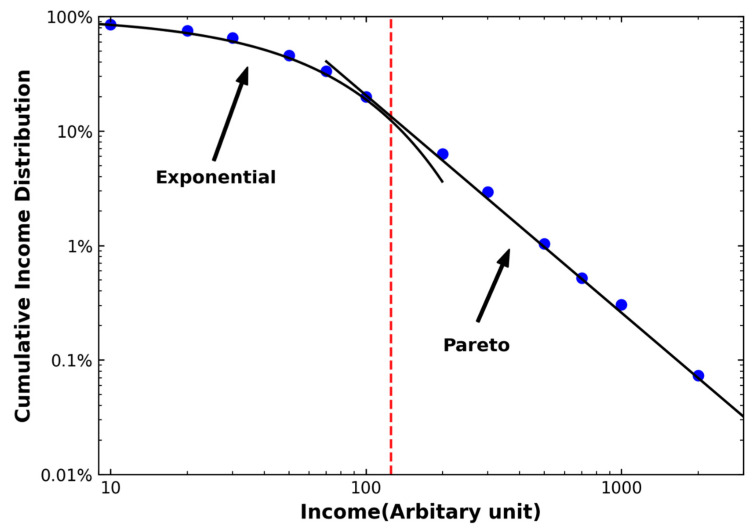
The income distribution function exhibits a typical pattern across different income levels. While most incomes follow an exponential distribution, the high-income class follows Pareto’s law, characterized by a fat-tail distribution.

Figure 1 illustrates a typical income distribution function, where the cumulative income distribution is represented on the vertical axis. In this distribution, approximately 90% or less of the population follows an exponential distribution, while the top earners exhibit a power-law pattern. The specific value of the transition income, $w_{c}$, which distinguishes the exponential function from the power law, varies among different countries. Additionally, the values of the Pareto index α, effective wealth temperature $T_{w}$, and transition income $w_{c}$ are not universal, as they vary across countries and years.

## 3. Measures of Wealth Inequality

There are multiple methods available to evaluate income inequality. One commonly used approach involves sorting a country’s income in ascending order and dividing it into intervals. The ratio between the upper and lower intervals serves as an indicator of income inequality. Representative indicators include the decile distribution ratio, quintile scale, and percentile ratio, which all utilize distribution ratios to measure income inequality. Additionally, various indices such as the Gini coefficient, Hoover index, Kolkata index, Theil index, Atkinson index, and Pietra index are employed to assess income inequality. Let us explore some of these inequality indices [58,59,60,61,62,63]. 

The decile distribution ratio (DDR) is an index used to quantify the income distribution ratio within a country [58,63]. It involves sorting the income of all households in ascending order and dividing them equally into ten groups. The decile distribution ratio, denoted as R10, is defined as follows:

R10 = Income share of the bottom 40%/Income share of the top 20%.(5)

Regarding the decile distribution ratio (DDR), as the lower income levels rise, the DDR also increases. Consequently, a higher DDR value suggests a more equitable distribution of income, whereas a lower DDR indicates a higher degree of income inequality.

(6) $$0\;\left(unequal\right)\;\leq R10\;\leq 2\;\;\left(equal\right).$$

If the DDR value is 0.45 or higher, it signifies a favorable distribution state. A DDR value ranging between 0.35 and 0.45 suggests a state of normal distribution. Conversely, a DDR value below 0.35 indicates an unfavorable and unequal income distribution.

The quintile scale, denoted as R5, represents the ratio between the average income of individuals in the highest quintile and the average income of individuals in the lowest quintile. This ratio is calculated when the total population is divided into five groups based on equalized individual income [58,63]. The quintile scale illustrates the extent to which income in the fifth quintile surpasses the income in the first quintile. For instance, a fifth quintile multiplier of 5.85 indicates that the income in the fifth quintile is 5.85 times higher than the income in the 1st quintile.

R5 = Income share of the top 20%/Income share of the bottom 20%.(7)

The decile scale measures the ratio of the average income between individuals in the 10th decile and the 1st decile. To achieve equalization, individual incomes are sorted in ascending order and divided into equal parts, known as quantiles. Each quantile has an upper limit denoted as POO [58,63]. The ratio of these upper limits, POO, serves as the indicator for percentile rates. For example, the percentile rate P90/P10 represents the ratio of the upper ninth percentile to the upper first percentile. P40 represents the upper limit of the 4th quartile based on the 10th quartile, while P20 represents the upper limit of the 2nd quartile based on the 5th quartile. When evaluating income inequality using percentiles, the ratios P50/P10 or P90/P50 are commonly employed. In this context, P50 refers to the median income of the entire population. Therefore, P50/P10 signifies the ratio of the median income to the upper limit of the first decile, whereas P90/P50 represents the ratio of the upper limit of the ninth decile to the median income.

Ratios serve as straightforward and informative indicators of inequality, offering advantages such as easy computation and intuitive understanding. Among these measures, decile dispersion ratios, including the 20:20 ratio and the Palma ratio, are widely utilized [58,63]. The 20:20 ratio compares the wealth of the top 20% of individuals in a population with the wealth of the bottom 20% of individuals. It provides a clear representation of the wealth disparity between the richest and the poorest, measuring the multiple by which the rich exceed the poor in terms of wealth. On the other hand, the Palma ratio compares the share of wealth held by the top 10% of individuals with the share of wealth held by the bottom 40% of individuals. This ratio, introduced by Palma, is based on the empirical observation that the combined share of these two groups roughly equals the share of the remaining group, often referred to as the middle class.

**Figure 2:**
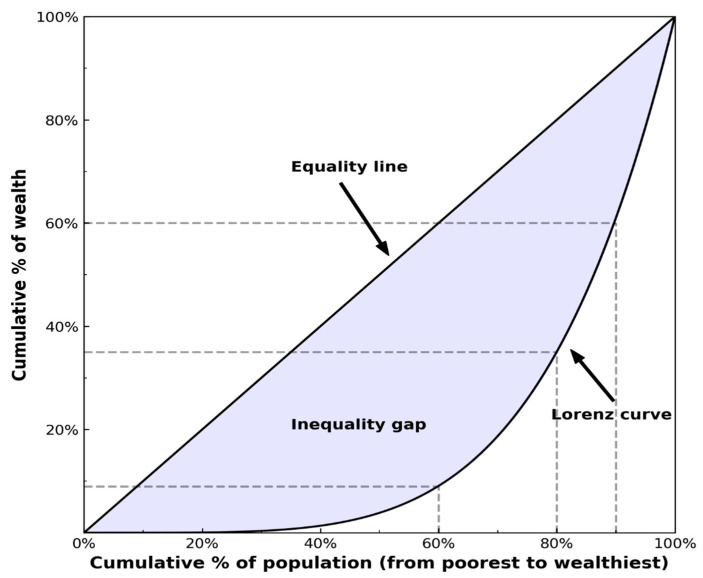
The Lorenz curve illustrates the cumulative percentage of income or wealth on the horizontal axis, while the vertical axis represents the corresponding cumulative percentage of the population. The data are arranged in ascending order based on income or wealth, starting from the lowest and progressing towards the highest.

The Gini coefficient is widely employed as a metric for assessing income, consumption, and wealth inequality [58,63]. It can be computed using the Lorentz curve, which is constructed by arranging the entire population in ascending order of income and setting the total population to 100. In Figure 2, the horizontal axis represents the cumulative population ratio, while the vertical axis represents the cumulative income ratio, with 100 representing the total cumulative income of the population. The Lorenz curve is defined as the line connecting these two ratios and provides a precise visual depiction of income distribution and inequality. To calculate the Gini coefficient, the area between the Lorenz curve and the line of perfect equality is divided by the area of the triangle below the line of equality. This coefficient ranges from 0 to 1, where 0 indicates perfect equality and 1 represents perfect inequality.

Figure 2 depicts the distribution of accumulated wealth across the population under the assumption that all individuals possess an equal amount of wealth (i.e., $p\left(w\right)=\delta \left(w-w_{o}\right)$). In such a scenario, the distribution takes the shape of a straight line, often known as the equality line. On the other hand, the Gini coefficient can be calculated using the following formula when a variable $y_{i}$ (i=1,⋯,N) representing economic indicators like personal income or GDP is arranged in ascending order:

The Gini index is defined as [58,63]

(8) $$Gini=\frac{2}{N-1}\sum_{i=1}^{N-1}\left|F_{i}-Q_{i}\right|,$$

where

(9) $$F_{i}=\frac{i}{N},$$

(10) $$Q_{i}=\frac{\sum_{j=1}^{i}y_{i}}{\sum_{j=1}^{n}y_{i}}.$$

The Hoover index can be computed by subtracting the percentage of people receiving less than their equal share (i.e., less than the national mean income) from their percentage of the national income. The Hoover index is defined as [59,62].

(11) $$h=\frac{1}{2}\frac{\sum_{i}^{}\left|y_{i}-\bar{y}\right|}{\sum_{i}^{}y_{i}}.\;$$

The Hoover index represents the percentage of total wealth necessary to achieve perfect equality. In Figure 2, the Hoover index is determined by the length of the vertical line drawn between the equality line and the Lorenz curve. This vertical line has the greatest length among all possible vertical lines. The Hoover index is calculated as the ratio of total wealth to above-average wealth. A Hoover index between 0 and 1 indicates a perfectly equal income distribution, where h = 0 represents perfect equality and h = 1 signifies perfect inequality.

Ghosh et al. proposed a new wealth inequality index known as the κ-index or Kolkata index [59]. The κ-index is computed from the intersection (k, 1 − k) of the reverse diagonal with the Lorenz curve. The κ-index represents the fraction (1 − k) of people who earn more than the fraction k of people in the country or society.

## 4. Models of Income and Wealth Distribution

Kinetic exchange models for money or wealth have been introduced and simulated using agent-based models in various situations. The fundamental model involves the random exchange of money between two agents selected randomly. Since the initial introduction of the kinetic exchange model, researchers have developed modified versions that incorporate features such as debts, taxation, and saving propensity [5,6,7,8,9,10,11,12,13,14,15,16,17,18,19,20,21,22,23,24,25,26,27,28,29,30,31,32,33,34,35,36,37,38,39,40,41,42,43]. Now, let us delve into the topic of kinetic exchange models for money or wealth.

### 4.1. Stochastic Multiplicative Process

Bouchaud and Mezard introduced a simple model for wealth evolution that demonstrates the phenomenon of wealth condensation [20]. The model considers an agent with wealth $w_{i}\left(t\right)$ at time t and introduces a stochastic dynamical equation to describe the evolution of wealth:

(12) $$\frac{dw_{i}}{dt}=\overset{}{\underset{j\neq i}{\sum }}J_{ij}w_{j}-\overset{}{\underset{j\neq i}{\sum }}J_{ji}w_{i}+w_{i}\eta_{i}\left(t\right),$$

where $\eta_{i}\left(t\right)$ is a Gaussian random noise with a mean m and the variance $2\sigma^{2}$. The equation of wealth evolution introduced by Bouchaud and Mezard takes into account the effect of noise on the variation of wealth for each agent. The interaction strength $J_{ij}$ determines the amount of wealth transferred from agent j to agent i through economic activities. To simplify the model, the authors assume a constant interaction strength $J_{ij}=J/N$ for all i≠j where N is the total number of agents.

(13) $$\frac{dw_{i}}{dt}=\left(\eta_{i}\left(t\right)-m-\sigma^{2}\right)w_{i}+J\left(1-w_{i}\right),$$

where $\eta_{i}\left(t\right)$ represents Gaussian random noise with a mean m and variance $2\sigma^{2}$. The equation captures the effect of noise on the wealth variation of each agent. The interaction strength $J_{ij}$ determines the amount of wealth transferred from agent j to agent i through economic activities. 

By further simplifying the model, the authors derive the wealth distribution as:

(14) $$p\left(w\right)=C\frac{e^{-\left(\mu -1\right)/w}}{w^{\mu +1}},$$

where $\mu =1+J/\sigma^{2}$, and the normalization constant is given as $C={\left(\mu -1\right)}^{\mu }/\Gamma \left(\mu \right)$. In the high wealth range, the wealth distribution follows a power law of the form $p\left(w\right)\sim w^{-\left(\mu +1\right)}$, where the Pareto exponent $\mu =1+J/\sigma^{2}$, which depends on the interaction strength and the variance of the random noise.

### 4.2. Boltzmann Distribution of Wealth

The distribution of wealth is influenced by the economic activities of individuals engaging in money or asset exchanges. In real-world economic transactions, wealth is transferred through the trading of goods or the purchase of services, rather than direct money exchange. When the total amount of money is conserved within a closed system, the distribution of money resulting from random money exchanges follows the Boltzmann distribution. In an actual economic system where the total amount of money is not conserved and economic activity is ongoing, the distribution of money does not reach equilibrium. However, if the total amount of money in the market remains relatively constant over a period and economic activity is active, the distribution of money will eventually reach a steady state [64,65].

To address the issue of money conservation, Dragulescu and Yakovenko proposed a money exchange model in which the total amount of money is conserved [21]. In this model, two agents, denoted as i and j, each possessing money ($m_{i}$ and $m_{j}$, respectively), exchange a portion of their money with each other, akin to gas molecules exchanging energy during collisions. The rules governing this money exchange process are as follows:

(15) $$m_{i}^{\prime }=m_{i}+\Delta m$$

(16) $$m_{j}^{\prime }=m_{j}-\Delta m,$$

where ∆*m* > 0 and the amount of money owned by each agent, $m_{i}$ and $m_{j}$, is non-negative. The conservation condition of money before and after a transaction is expressed as $m_{i}^{\prime }+m_{j}^{\prime }=m_{i}+m_{j}$. As the system reaches a steady state, the distribution function of money can be derived using the Boltzmann kinetic equation. The evolution equation for the money distribution function, $P\left(m\right)$, is given as [12].

(17) $$\frac{dP\left(m\right)}{dt}=\iint [T(m-\Delta m,\;m_{}^{\prime }+\Delta m\;\to m,m_{}^{\prime })P\left(m-\Delta m\right)P\left(m_{}^{\prime }+\Delta m\right)\;-T\left(m,m_{}^{\prime }\to m-\Delta m,m_{}^{\prime }+\Delta m\right)P\left(m\right)P\left(m_{}^{\prime }\right)]dm_{}^{\prime }d\Delta m,\;$$

where $T\left(m,m_{}^{\prime }\to m-\Delta m,m_{}^{\prime }+\Delta m\right)$ represents the probability of money transition. Money Δm per unit time is transferred from the agent with money m to the agent with money m′. In Equation (5), the first term is $\left(m_{}^{\prime }-\Delta m,m_{}^{\prime }+\Delta m\to m,m_{}^{\prime }\right)$, indicating an increase in the probability $P\left(m\right)$ due to the transfer of money. On the other hand, the second term $\left(m,m_{}^{\prime }\to m-\Delta m,m_{}^{\prime }+\Delta m\right)$ results in a decrease in probability $P\left(m\right)$ due to the transfer of money. When the exchange of money reaches a steady state, $dP\left(m\right)/dt=0$. In an equilibrium state where the economic system exchanging money satisfies the detailed balance condition, the transition probability can be expressed as follows.

(18) $$T\left(m-\Delta m,\;m_{}^{\prime }+\Delta m\;\to m,m_{}^{\prime }\right)=T\left(m,m_{}^{\prime }\to m-\Delta m,m_{}^{\prime }+\Delta m\right).$$

Therefore, the equilibrium condition is given as

(19) $$P\left(m\right)P\left(m_{}^{\prime }\right)=P\left(m-\Delta m\right)P\left(m_{}^{\prime }+\Delta m\right).\;$$

The solution of this equation is given as

(20) $$P\left(m\right)=Cexp\left(-\beta m\right),\;$$

where C is the normalization constant and β corresponds to the temperature variable in the money exchange market. The distribution of money in the equilibrium follows the exponential function.

### 4.3. Kinetic Exchange Models of Wealth

**Figure 3:**
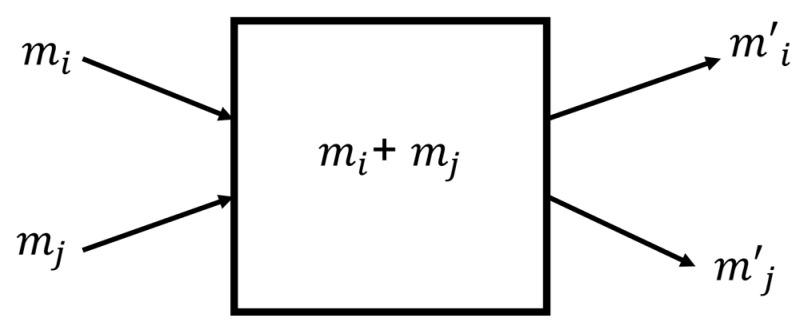
The dynamics of money in kinetic exchange. In this process, two agents pool all their money into a common box and then redistribute it amongst themselves.

Since 2000, research has been conducted on the distribution of wealth in the money exchange model. Dragulescu and Yakovenko discovered that the distribution of wealth in the model described by Equations (3) and (4) follows an exponential distribution [21]. In this model, two agents exchange a specific amount of money, Δm. If we set $\Delta m=r\left(m_{i}+m_{j}\right)$, where r is a random number uniformly distributed between 0 and 1, the distribution of money in the steady state follows an exponential distribution [15,21]. This model is represented as

(21) $$m\prime_{i}=\varepsilon_{ij}(m_{i}+m_{j}),$$

(22) $$m\prime_{j}=\left(1-\varepsilon_{ij})(m_{i}+m_{j}\right),$$

where $\varepsilon_{ij}$ is a random variable uniformly distributed between zero and one. We represent the exchange dynamics in Figure 3.

The money exchange model can accommodate the inclusion of debt, which means that an agent’s money can take on negative values [21,66]. To address this, Dragulescu and Yakovenko proposed a model that introduces a lower bound on an agent’s debt, ensuring that the total amount of money remains conserved as the debt is borrowed from a reservoir. This lower bound constraint ensures that all agents’ money satisfies the condition $m_{i}>-m_{d}$. Models that incorporate debt exhibit an exponential distribution function, encompassing the range of negative wealth. However, Xi et al. made a notable discovery that in cases where there is no limit on individual debt but a limit on system-wide debt, the distribution of money follows an asymmetric Laplace distribution [66].

### 4.4. Money Exchange Models with Saving

When individuals engage in economic activities, they typically allocate a portion of their money for savings instead of spending it all at once. To accommodate this behavior, a modification can be made to the money exchange model to incorporate savings. Ispolatov et al. introduced a multiplicative asset exchange model where a fraction of an agent’s money is exchanged, denoted as $\Delta m_{i}=\gamma m_{i}$. The total amount of money in the system remains conserved, and the distribution function of money in this model follows a gamma distribution function, given by $p\left(m\right)=cm^{\beta }e^{-m/T_{m}}$. The exponent of the power law, β, is determined by $\beta =-1-ln2/\ln\left(1-\gamma \right)$, and $T_{m}$ represents the money temperature. Additionally, Chakraborti and Chakrabarti developed a model in which agents exchange a certain percentage of their own money. Specifically, agent i, with money $m_{i}$, sets aside a percentage $\lambda m_{i}$ of their money and exchanges the remaining $\lambda \left(1-m_{i}\right)$ in the money exchange process [21]. The money exchange model proposed by Chakraborti and Chakrabarti is as follows:

(23) $$m\prime_{i}=\lambda m_{i}+\left(1-\lambda \right)\epsilon \left(m_{i}+m_{j}\right),$$

(24) $$m\prime_{j}=\lambda m_{j}+\left(1-\lambda \right)\left(1-\epsilon \right)\left(m_{i}+m_{j}\right).$$

The saving propensity is represented by λ, and the asymmetric money distribution ratio is denoted by ϵ, which is a random number between 0 and 1. In their model, the distribution function of money follows a gamma distribution with an exponent of $\beta =3\lambda /\left(1-\lambda \right)$ [17,39,40,67,68].

(25) $$m\prime_{i}=\lambda_{i}m_{i}+\epsilon \left(1-\lambda_{i}\right)\left(m_{i}+m_{j}\right),$$

(26) $$m\prime_{j}=\lambda_{j}m_{j}+\left(1-\epsilon \right)\left(1-\lambda_{j}\right)\left(m_{i}+m_{j}\right).$$

Chatterjee et al. discovered that when the saving propensity is randomly assigned, the distribution of money follows a power law [37].

(27) $$m\prime_{i}=\lambda_{i}m_{i}+\epsilon \left[\left(1-\lambda_{i}\right)m_{i}+\left(1-\lambda_{j}\right)m_{j}\right],$$

(28) $$m\prime_{j}=\lambda_{j}m_{j}+\left(1-\epsilon \right)\left[\left(1-\lambda_{i}\right)m_{i}+\left(1-\lambda_{j}\right)m_{j}\right].$$

They randomly assigned a saving propensity $\lambda_{i}$ to each agent, using a uniform distribution between 0 and 1. In the steady state, the distribution function of money was observed to follow a power law, characterized by $p\left(m\right)\sim m^{-\left(1+\nu \right)}$. Through Monte Carlo simulations, they determined that the power function exponent was ν=1.02. Additionally, Patriaca et al. discovered that the power function was predominantly influenced by agents with saving propensities λ close to 1 [33,41,69].

### 4.5. Money Exchange Models with Tax

Taxes play a vital role in addressing wealth inequality by redistributing wealth across society. Governments, whether national or local, collect taxes to fund various public services and social infrastructure, and provide support to low-income households [70,71,72,73,74,75,76,77]. Dragulescu and Yakovenko proposed a model that incorporates a tax on money exchange, which revealed that the imposition of taxes altered the wealth distribution function, deviating from the Boltzmann–Gibbs distribution and approximating the gamma distribution function. Additionally, de Oliveira proposed an annual random wealth multiplicative model for taxation, where the distribution of wealth depends on how taxes are imposed. If the tax burden falls disproportionately on the poor, wealth tends to condense in one agent, while taxing the rich more can lead to continued wealth evolution [12,21,72].

Fernandes and Tempere introduced a kinetic model in which agents on a two-dimensional grid exchange wealth with neighboring agents. Their research highlighted that the most effective mitigation of inequality occurred when the wealth tax rate was approximately 40% [73]. Furthermore, Banzhf developed an agent-based model to examine the implications of income tax and wealth tax. [74] In the case of a flat income tax, where individuals are taxed at a constant rate from their income at regular intervals, two scenarios were explored: one where all collected taxes are equally distributed among all individuals and another where taxes are allocated to the lowest income earners. The income of an agent in the tax-inclusive model is adjusted by the tax amount during each cycle, determined by the tax collection rate (r) and tax cycle (T).

(29) $$m_{i}\left(t+1\right)=m_{i}\left(t\right)-r[m_{i}\left(t\right)-m_{i}\left(t-T\right)]\;\mathrm{if}\;mod\left(t,T\right)=0.$$

The total amount of taxes collected from all individuals is represented as $M\left(t\right)$. In the flat income tax model, when the collected taxes are distributed equally among all individuals, the income of each individual is adjusted according to the following equation:

(30) $$m_{i}\left(t+1\right)=m_{i}\left(t\right)+\frac{M\left(t\right)}{N},$$

where N denotes the total number of individuals. However, distributing taxes equally among all individuals often leaves a significant number of them in poverty. Conversely, redistributing the taxes to a specific quintile of earners helps reduce income inequality.

### 4.6. Non-Conservative Kinetic Exchange Models of Wealth

In real economic systems, money and wealth are not conserved. Societies experience dynamic changes in population, and prices and wages constantly fluctuate. Traditionally, many money exchange models have assumed the conservation of the total amount of money. However, economic systems do not adhere to the conservation of total money, requiring the development of models that acknowledge the non-conservation of wealth as well. While local or short-term conservation of money may occur, it is not sustained on a broader or long-term scale. Recent research has been proposing non-conserved money exchange models [78,79,80,81,82,83,84,85,86,87,88,89,90,91,92]. One such model, presented by Bouchaud and Mezerd, represents the inflow of wealth as a stochastic multiplicative process, resulting in exponential growth of average wealth [20]. Slanina also proposed a wealth exchange model for open economy systems, where wealth grows and exchanges take place [87].

(31) $$w_{i}\left(t+1\right)=\left(1+\epsilon -\beta \right)w_{i}\left(t\right)+\beta w_{j}\left(t\right),$$

(32) $$w_{j}\left(t+1\right)=\beta w_{i}\left(t\right)+\left(1+\epsilon -\beta \right)w_{j}\left(t\right).$$

In this model, the parameter β represents wealth exchange, while ϵ>0 denotes the inflow of wealth from external sources. Consequently, the model considers the system as open, leading to wealth growth. The distribution of wealth exhibits a power-law pattern in its tail, with the exponents of the power-law determined by the parameters β and ϵ. Cordier et al. introduced a wealth exchange model where wealth increases with multiplicative noise, and individuals exchange a certain amount of wealth [88]. They derived the asymptotic behavior of the Fokker–Planck equation for wealth distribution and observed that the long tail of the distribution follows a power law, with the exponents depending on the strength of wealth exchange and the variance of the random noise.

Quevedo and Quimbay proposed a non-conservative wealth exchange model that incorporates the savings of production goods [80]. Their research revealed that the wealth distribution function follows a gamma distribution, and when the variable determining the saving rate is large, the Gini index exceeds 0.5. In the range of large negative values, the power function exponent of the gamma distribution was determined by the agents’ exchange aversion variables.

### 4.7. Kinetic Exchange Models with Wealth Condensation

Wealth condensation has been observed in wealth exchange models with multiplicative wealth growth [89,90,91,92,93,94,95,96,97,98,99,100,101,102,103,104,105,106]. Burda et al. conducted a study on a closed macroeconomic system, where a substantial portion of the total wealth accumulates in the hands of a single individual [94]. Pienegonda et al. introduced a model of wealth redistribution with conservative exchanges, focusing on the dynamics occurring at the poorest agent to mimic extreme wealth dynamics [95]. They observed a form of wealth condensation where only a small number of rich agents remain stable over time [95].

Moukarzel et al. proposed a random asset exchange model, where at each time step, two agents bet for a fraction of the poorest agent’s wealth [88,99]. They obtained a phase diagram illustrating the occurrence of wealth condensation when the poorest agent wins the bet with a certain probability. Wealth condensation has been observed in various yard-sale models of asset exchange [100,101,102,103,104,105,106]. Liu et al. developed an agent-based yard-sale (YS) model that incorporates wealth inequality [84]. They observed the emergence of wealth condensation when the transferred wealth allocation was biased towards agents with higher wealth.

Saif and Gade proposed an asset exchange model that combines the yard-sale (YS) model and the theft–fraud (TF) model, incorporating a mixed exchange [100,104]. In the YS model, two agents exchange an amount of wealth $\Delta m=\alpha \min\left[m_{i}\left(t\right),m_{j}\left(t\right)\right]$ according to Equations (13) and (14). In the TF model, the money exchange is a fraction of the wealth of the losing player, with $\Delta m=\alpha m_{j}\left(t\right)$ if j is the loser. The parameter α is a random number within the interval [0, 1]. They observed wealth condensation in the YS model. In the pure TF model, the wealth distribution follows an exponential distribution. However, in the mixed strategic model combining the YS and TF models, the wealth distribution follows a power-law distribution.

Ichinomiya studied the modified Bouchaud–Mezard model in a random network and observed wealth condensation, characterized by the divergence of wealth variance [98]. Cui et al. observed wealth condensation in the bequeathed model, where a fraction of the wealth is transferred to a single heir or equally distributed among all heirs upon a person’s death [104]. In the wealth transfer to a single heir, the wealth distribution exhibited wealth condensation, where a single individual accumulates all the available wealth.

## 5. Discussion

Wealth inequality has become an increasingly critical issue in the economy, necessitating a comprehensive exploration of various models concerning money and wealth exchange. This review paper also introduces measures that effectively quantify wealth inequality, including well-known indices such as the Gini coefficient, the Hoover index, and the Kolkata index. Through an analysis of cumulative wealth distribution based on income distribution across different countries, a dual distribution function is revealed, encompassing both an exponential and power-law function.

To delve into the wealth distribution function, this paper examines studies on stochastic wealth dynamics and wealth exchange models. Stochastic wealth dynamics express the wealth distribution as a combination of a power distribution and an exponential function. In the kinetic wealth exchange model, two agents engage in exchanging their wealth, either in its entirety or partially. Multiple models have been proposed, incorporating various strategies for wealth exchange, such as debt inclusion, saving propensity, and taxation.

In cases of random wealth exchange, the wealth distribution follows an exponential function. However, when individuals save a portion of their wealth and exchange the remaining, the distribution exhibits a power law. Furthermore, a model incorporating a specific tax on wealth exchange has been proposed. Considering the non-conservation of money and wealth in a market economy, models have also been presented where money is not conserved. For instance, in the yard-sale model, wealth condensation occurs when a poorer agent exchanges a portion of their wealth with a wealthier agent.

The kinetic exchange models ignore to consider fundamental features of the real world, such as the absence of product exchange and a market for trading commercial products and services. To develop a more comprehensive understanding of economic dynamics, it is crucial to extend these models and integrate key concepts from real economics. Furthermore, the role of capital is of paramount importance in shaping income inequality. Considering the diverse and heterogeneous nature of the market and the broad spectrum of agents involved will be vital in addressing these complexities. Given that wealth is transferred through the exchange of products and services in real economic systems, it is imperative to conduct further research on wealth exchange models that consider barter or exchange in kind.

Another avenue for expanding the study of wealth dynamics involves utilizing Monte Carlo simulations and agent-based models. These approaches offer valuable insights into how wealth evolves over time. Within the heterogeneous agent-based model, we can incorporate various types of agents, including people, products, labor, capital, and services. This enriched model allows for a more comprehensive analysis of wealth dynamics. In the Monte Carlo simulation of the kinetic exchange model, random pairs of agents engage in money or wealth exchange, following predefined rules for transfer. This simulation method provides a dynamic perspective on wealth dynamics. The combined use of Monte Carlo simulations and agent-based models contributes to a deeper understanding of wealth dynamics, shedding light on complex interactions and patterns within economic systems.

## Data Availability

Data sharing is not applicable.
